# Dermatologists' Way of Informative Content on Dermatology and Cosmetology on Social Media

**DOI:** 10.1111/jocd.70864

**Published:** 2026-04-15

**Authors:** Kainat Zulfiqar, Ahmad Furqan Anjum, Iqra Moatter Nurie

**Affiliations:** ^1^ Department of Internal Medicine Rawal Medical College Islamabad Islamabad Pakistan; ^2^ Department of Internal Medicine Shaikh Khalifa Bin Zayed Al Nahyan Medical and Dental College Lahore Lahore Pakistan; ^3^ Department of Internal Medicine Graduate of Baqai Medical University Karachi Karachi Pakistan

Dear Editor,

I would like to applaud the authors of the article titled “Dermatologists' Way of Informative Content on Dermatology and Cosmetology on Social Media,” published in the Journal of Cosmetic Dermatology [1]. The study provides a timely and helpful insight into the utilization of social media sites by dermatologists in Turkey to publish informative materials, especially in the field of dermatology and cosmetology. In the age of online misinformation, the attempt of the authors to shed light on the intentions of dermatologists to reach the masses through online resources is useful and well‐deserved. The issue is particularly relevant in the context of the growing impact of social media on the health behaviors of the entire population and treatment choices. The authors should be appreciated as they have given an openness to an important debate. Nevertheless, they have some limitations in the study.

The study uses self‐reported data from dermatologists; its data collection lacks the use of objective content analysis or an observational record of the behavior of actual social media usage. Such use of self‐reported data may introduce biases like social desirability or memory bias, thus misrepresenting the nature of informative content posted by dermatologists. For example, in an analysis of Instagram, Dorfman et al. (2020) [2] revealed that the most popular posts were self‐promoting rather than educational, contrary to the claims of many providers of being informative. Therefore, future research must include content analysis to ensure that the self‐reported information convincingly establishes behavioral trends. Additionally, the study is also limited to the perspectives and practices among dermatologists, thus failing to evaluate the impacts and responses of dermatologic content on the population. This constrains the study to analyze the real‐world influence of the educational material. For example, Vraga et al. (2022) [3] discussed YouTube skin cancer videos, which revealed that there was little comprehension and belief in unverified advice, creating misperceptions among the public. Future research should incorporate both surveys of the general population and analytics of user engagement to evaluate how people interact. This will show how subsequent actions are influenced by dermatological materials.

Furthermore, the study did not differentiate between personal, professional, or institutional accounts. The lack of differentiation suppresses important questions about professionalism, content control, and adherence to ethical or institutional directives on medical communications. For example, as explained by Ventola et al. (2014) [4], neglecting to distinguish between personal and professional use of social media among care providers resulted in situations of breaching patient confidentiality and reputational damage. Future research should clearly differentiate between the types of accounts in surveys and assess how the purposes of creating platforms or accounts influence the style of content and compliance with regulations. In addition, the paper gives little attention to patient consent and does not mention wider ethical issues, including product endorsements, potential conflicts of interest, and privacy protection, which risks underestimating the ethical aspects of using medical social media. There is potential to find commercial incentives or court definitions of negligence. For example, Helou et al. (2023) [5] warned that most clinicians on social media endorsed products or treatments without reporting any affiliations, which raised some ethical and trust issues. Consequently, future studies should broaden the ethical consideration using systematic tools or checklists to assess promotional transparency, informed consent, and content verifiability.

Moreover, this research is restricted to Turkey and fails to take into consideration the differences in social media literacy, cultural norms, and dermatological practices in other areas. This makes it difficult to apply the findings in international contexts or even within populations that live in the same country. For example, different regions have been described to show vast differences in credibility and engagement with dermatology content on TikTok and enforcement (Nigro et al. 2025) [6]. Hence, future research should be aware of regional differences and achieve a larger application and generalization of results.

Although this study yields valuable information about the interest of dermatologists in using social media, the specified limitations should be addressed to strengthen the rigor, applicability, and impact of the research. The next step in research involving a more eclectic methodology and a more diverse potential participant population will be critical in helping us get a better grasp of this changing landscape of dermatology and digital communication.

## Author Contributions

Kainat Zulfiqar contributed to the conception, design, and drafting of the letter to the editor, and played a primary role in the overall coordination and execution of the work. Ahmad Furqan Anjum and Iqra Moatter Nurie participated in the critical revision of the letter for important intellectual content and assisted in the final approval of the version to be submitted. All authors meet the ICMJE criteria for authorship, including having read and approved the final manuscript.

## Funding

The authors have nothing to report.

## Ethics Statement

The authors have nothing to report.

## Conflicts of Interest

The authors declare no conflicts of interest.

## Linked Articles

This article is linked to Ünal and Demirel papers. To view this article, visit https://doi.org/10.1111/jocd.70148.

## Data Availability

Data sharing not applicable to this article as no datasets were generated or analysed during the current study.
